# Concurrent sarcoidal granulomas and melanoma micrometastasis in a sentinel node – a case report

**DOI:** 10.1080/23320885.2019.1694412

**Published:** 2019-12-05

**Authors:** Hannah Trøstrup, Nille Behrendt, Anne Mellon Mogensen, Iselin Saltvig, Phillipe Claus Bandier, Jørgen Hesselfeldt, Jette B. Løvenwald

**Affiliations:** a Department of Plastic Surgery and Breast Surgery, Zealand University Hospital, Roskilde, Denmark;; b Department of Pathology, Zealand University Hospital, Roskilde, Denmark

**Keywords:** Sentinel node, sarcoidosis, micrometastasis, melanoma, granulomas

## Abstract

Incidental findings of non-caseating granulomas and metastasis in sentinel lymph nodes are rare but cause clinical challenges. We report a case of coinciding unexpected asymptomatic lymphoid sarcoidosis and a micrometastasis in a sentinel node of a patient, who was newly diagnosed with 2.0 mm thick melanoma on the left calf.

## Introduction

Sarcoidosis is a relatively uncommon systemic autoimmune disease characterised by the development of non-caseating granulomas in multiple organ systems, mostly the lungs and the lymph nodes. Clinical symptoms are fatigue, weight loss, and pulmonary symptoms. Sarcoidosis predispose individuals to cancer [1] and melanoma and non-melanoma skin cancer [2]. Current adjuvant check point inhibitors (CPI) are a breakthrough in the treatment of advanced melanoma. Several cases of CPI-induced sarcoid granulomatosis has been reported [3–5] indicating a link between metastatic melanoma and sarcoidosis. Concurrent sarcoid granulomas and metastases of melanoma in lymph nodes of CPI-naïve patients are rare. According to one study including 1,199 patients with melanoma, the prevalence of simultaneous sarcoidosis and melanoma is 0.58% [6]. Another paper describes two cases of melanoma in 80 patients diagnosed with sarcoidosis [7].

Sarcoidosis is caused by an aberrant immune response towards an unknown agent, yielding repeated cycles of Tumour Necrosis Factor-α production and infiltrating T-helper-1 cells and macrophage. The interaction between these pleomorphic manifestations in the lymph nodes and predisposition to metastatic melanoma is unknown.

## Case

**Figure 1.**
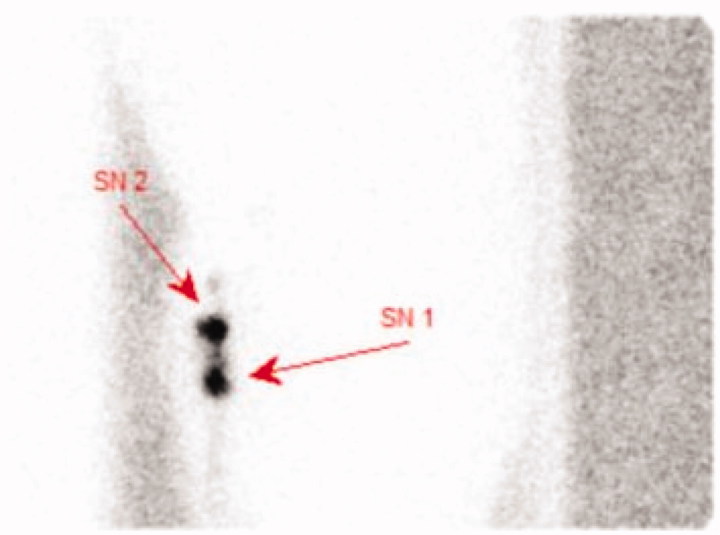
Lymphoscintigraphy displaying two sentinel nodes in the left groin.

A 43-year old healthy male with no familiar disposition of or previous history of melanoma or symptoms of pulmonary, systemic, or cutaneous sarcoidosis was referred with a 2.0 mm thick, intermittently bleeding superficially spreading malignant melanoma on the left calf. AJCC (7th edition) stage was pT2a. No lymhadenopathy was found by clinical examination. A 2.0 cm margin of excision to fascia was performed. The sentinel node was located by preoperative lymphoscintigraphy (Figure 1). Blue dye staining was then injected intradermally at the primary tumour site and by use of a hand held γ probe, the sentinel node was confirmed and surgically removed.

**Figure 2.**
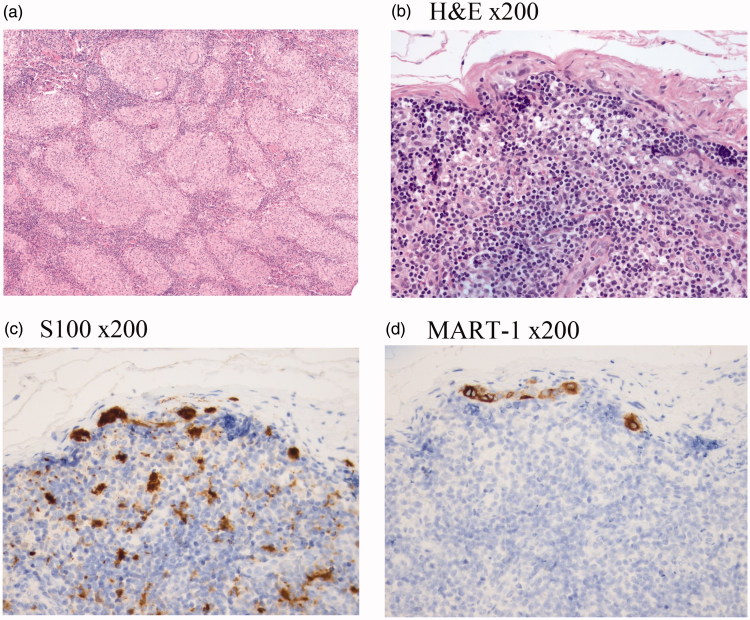
(**a**) Abolished microarchitecture and epitheloid granulomas of the sentinel node from the left groin. Hematoxylin and eosin staining (H&E) x50. (b–d) Micrometastasis (partly single cell spread) in the lymph node. Pictures represent the same section in the lymph node.

**Figure 3.**
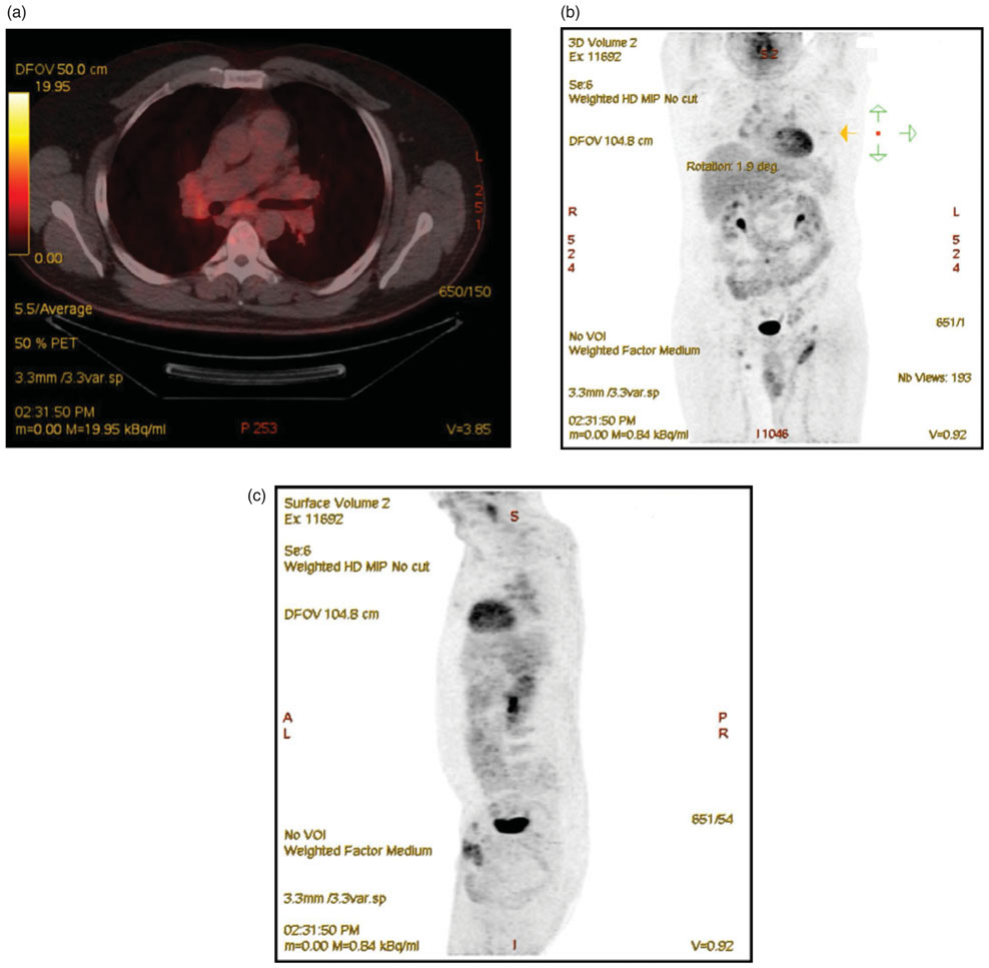
^18^-Fluorodeoxyglucose positron emission tomography (PET) and Computed Tomography (CT) in transverse (a), coronal (b) and sagittal (c) images. The transverse image (a) shows multiple active lymph nodes in the mediastinum.

Surprisingly, concurrent sarcoidal granulomas (Figure 2(a)) and a micrometastasis of melanoma (Figure 2(b–d)) was described in the same sentinel lymph node.^18^ Fluorodeoxyglucose positron emission tomography and Computed Tomography (^18^FDG-PET-CT) revealed multiple metabolically active mediastinal lymph nodes (Figure 3(a–c)), above and below the diaphragm, along the left iliacal vessels and in both groins. Histopathology confirmed the diagnosis lymphoid sarcoidosis and excluded metastatic melanoma. The patient will attend a five year follow up with an interval of three months and control PET-CT and ultrasound scans.

## Discussion

A search on Pub Med including the search words ’Sarcoidosis’ and ’Melanoma’ disclosed 174 hits until February, 2019, with most cases describing induction of sarcoidosis following immunotherapy for melanoma. Eleven of the 174 hits described case reports (8 articles) or reviews (3 articles) on the topic. One article was omitted due to foreign language. A total of fifteen case reports describing coincidence of sarcoidosis and metastatic melanoma were found (Table 1). In a systematic review based on a search on Pub Med from inception to April 2017, eight cases of sarcoidosis and melanoma were described [8]. In a case report and literature study by Beutler and Cohen, 17 out of 39 patients were diagnosed with sarcoidosis directly associated with melanoma, and in 12 of these 17 cases, melanoma preceded sarcoidosis [9]. A possible link between systemic sarcoidosis and haematological malignancies was described in 1972 by Brincker [1,10,11]. Sarcoidosis or granulomatous reactions are described in few case reports of patients with malignant melanoma [12,13]. A sarcoid-like reaction in the sentinel node draining a conjunctival melanoma has been described in one case study [14] and in a cutaneous nodule in proximity to a melanoma of a thigh [15]. In our case, we do not find melanoma-induced sarcoidosis plausible due to the very early state of dissemination of melanoma. In a patient with acral melanoma, micrometastatic melanoma cells and sarcoid granulomas were found in all regional lymph nodes [16]. In another case report on a 40-year old man who developed sarcoidosis and melanoma in a congenital nevus, the author speculates if sarcoidosis may act as a predisposing cause of melanoma [17]. There is a paucity of knowledge on pathophysiological immunological interactions causing simultaneous sarcoidosis and melanoma, which may cause diagnostic pitfalls and blur clinical treatment strategies for these patients. Besides being a potential side effect to current antineoplastic treatment strategies, sarcoidal granulomas may precede development of lymph node metastases of malignant melanoma in some predisposed patients.

**Table 1.** Case reports describing patients with sarcoidosis and melanoma.

## Consent

The patient gave written informed consent prior to publication of this case report.
